# Ischemic Preconditioning Promotes Autophagy and Alleviates Renal Ischemia/Reperfusion Injury

**DOI:** 10.1155/2018/8353987

**Published:** 2018-01-22

**Authors:** Ying Xie, Jing Xiao, Chensheng Fu, Zhenxing Zhang, Zhibin Ye, Xiaoli Zhang

**Affiliations:** ^1^Department of Nephrology, Huadong Hospital, Fudan University, Shanghai 200040, China; ^2^Shanghai Key Laboratory of Clinical Geriatric Medicine, Shanghai 200040, China

## Abstract

Autophagy is important for cellular survival during renal ischemia/reperfusion (I/R) injury. Ischemic preconditioning (IPC) has a strong renoprotective effect during renal I/R. Our study here aimed to explore the effect of IPC on autophagy during renal I/R injury. Rats were subjected to unilateral renal ischemia with or without prior IPC. Hypoxia/reoxygenation (H/R) injury was induced in HK-2 cells with or without prior hypoxic preconditioning (HPC). Autophagy and apoptosis were detected after reperfusion or reoxygenation for different time. The results showed that the levels of LC3II, Beclin-1, SQSTM1/p62, and cleaved caspase-3 were altered in a time-dependent manner during renal I/R. IPC further induced autophagy as indicated by increased levels of LC3II and Beclin-1, decreased level of SQSTM1/p62, and accumulation of autophagosomes compared to I/R groups at corresponding reperfusion time. In addition, IPC reduced the expression of cleaved caspase-3 and alleviated renal cell injury, as evaluated by the levels of serum creatinine (Scr), neutrophil gelatinase-associated lipocalin (NGAL), and kidney injury molecule-1 (KIM-1) in renal tissues. In conclusion, autophagy and apoptosis are dynamically altered during renal I/R. IPC protects against renal I/R injury and upregulates autophagic flux, thus increasing the possibility for a novel therapy to alleviate I/R-induced acute kidney injury (AKI).

## 1. Introduction

Acute kidney injury (AKI) is a common kidney disorder characterized by a rapid loss of renal function resulting in an accumulation of metabolic waste and an imbalance of electrolytes and body fluid [1]. Renal ischemia/reperfusion (I/R) injury is the most common risk factor for AKI. The tubular segments located within the outer stripe of the outer medulla, including the proximal tubules, are particularly sensitive to hypoxia and I/R injury because of their high rates of adenosine triphosphate consumption.

In response to injury, renal tubular cells activate a myriad of defense mechanisms. Studies have shown that autophagy is upregulated and plays an important role in AKI* in vitro* and* in vivo *[2]. Autophagy is an intracellular lysosomal degradation process involving the formation of a double-membrane structure known as an autophagosome, which sequesters long-lived proteins, damaged organelles, and malformed proteins and transfers them to lysosomes to be degraded by proteases, a process that yields nutrients that can be reused and energy that can be recycled [3]. Autophagy is induced under various stressful conditions including oxidant injury, cell starvation, growth factor deprivation, and I/R injury [4], and enhanced autophagy has been shown to protect renal tubular epithelial cells from I/R injury* in vitro* by attenuating apoptosis. Renal proximal tubule-specific autophagy-associated gene (Atg) knockout mice demonstrate autophagy inhibition and I/R injury sensitization compared to their wild-type littermates [5], confirming the renoprotective effect of autophagy in I/R-induced AKI.

Ischemic preconditioning (IPC), which was first described in the heart by Murry et al. [6], consists of brief periods of vascular occlusion. It has been reported to confer protection against subsequent I/R injury via endogenous protective mechanisms in various organs. This protection has been linked to decreased apoptosis [7], preservation of the ATP content, and overproduction of prosurvival and anti-inflammatory proteins [8, 9]. However, little is known about the effect of IPC on autophagy during renal I/R. In this regard, we monitored dynamic changes of autophagic flux in IPC-induced renoprotection utilizing the tandem RFP-GFP-LC3 adenovirus construct. Understanding the role of autophagy in IPC during renal I/R injury may provide a potential strategy for the clinical treatment of AKI in the future.

## 2. Materials and Methods

### 2.1. Design and Procedures of Animal Experiments

Sprague-Dawley male rats (8-9 weeks old; 230–270 g) were purchased from the Animal Center of Fudan University, Shanghai, China. Prior to the start of the experiment, the rats were kept at a constant temperature of 23 ± 2°C with 40%–60% relative humidity on a 12/12 h light/dark cycle and were given access to standard rodent chow and tap water ad libitum. All animal experiments described below were conducted in compliance with the Guide for the Care and Use of Laboratory Animals. Rats were randomized into the sham group, I/R group, IPC group, and IPC + I/R group, with eight rats in each group. The rats were anesthetized with 2% pentobarbital sodium (50 mg/kg, i.p.) and were kept on a heating pad to maintain their body temperature at 37°C. A midline laparotomy was then performed in which the abdominal cavity was fully exposed. In the I/R group, the left renal artery was isolated and clamped for 40 min using a nontraumatic artery clamp after a right nephrectomy. In the IPC + I/R group, rats were subjected to IPC prior to I/R, which consisted of four cycles of clamping the left renal artery for 8 min, separated by 5 min of reperfusion. The rats were sacrificed after reperfusion for 2 h, 6 h, 12 h, and 24 h to collect the blood and renal tissues for analysis. The rats in the IPC group underwent surgical manipulation and IPC without I/R. The rats in the sham group were subjected to surgical manipulation with no intervention.

### 2.2. Renal Function

Renal function was assessed by measuring serum creatinine (Scr) levels using a commercially available kit (Wako, Osaka, Japan).

### 2.3. Histology

Renal tissues were fixed with 10% formalin, embedded in paraffin, sectioned into 4 *μ*m thick slices, and stained with hematoxylin/eosin (H/E). An optical microscope (BX-51/DP-72; Olympus, Tokyo, Japan) was used to observe histological changes. Specimens were semiquantitatively scored by calculating the percentage of tubules showing cell necrosis, loss of the brush border, cast formation, and tubule dilatation, as follows: 0, none; 1, <5%; 2, 5–25%; 3, 25–75%; and 4, >75% [10]. At least ten consecutive fields in the corticomedullary junction and outer medulla were examined per section for each sample (200x magnification).

### 2.4. Western Blotting Analyses

The dissected renal tissues were homogenized in ice-cold lysis buffer for 15 sec. The samples were centrifuged at 10,000 r/min for 15 min, the supernatant was collected, and the protein concentrations were measured using the Bradford method. Approximately 30 *μ*g of protein extract per lane were loaded and separated using sodium dodecyl sulfate-polyacrylamide gel electrophoresis (SDS-PAGE) and then transferred to polyvinylidene fluoride membranes (Millipore, Billerica, MA, USA). The membranes were blocked with 5% nonfat milk and incubated with the primary antibodies for LC3B (1 : 500; Novus, Littleton, CO, USA), Beclin-1 (1 : 1000; Cell Signaling, Danvers, MA, USA), SQSTM1/p62 (1 : 1000, Abcam, Shanghai, China), cleaved caspase-3 (1 : 1000; Cell Signaling, Danvers, MA, USA), or GAPDH (1 : 5000; Sigma-Aldrich, St. Louis, MO, USA) in 0.5% BSA overnight at 4°C. After washing with TBST, the membranes were incubated with a horseradish peroxidase-conjugated goat anti-rabbit IgG antibody (1 : 5000, Jackson, West Grove, PA, USA) for 2 h. Signals were detected using the enhanced chemiluminescence (ECL) detection system (UVP Inc., Upland, CA, USA) and were quantified using scanning densitometry with the ImageJ analysis program (National Institutes of Health, MD, USA).

### 2.5. Immunohistochemistry

Formalin fixed kidney sections (4 *μ*m) were deparaffinized in xylene and rehydrated through a graded ethanol series to water. After blocking with 10% normal horse serum in PBS, the tissue was stained with rat NGAL and KIM-1 polyclonal antibody overnight at 4°C and then was stained with the Anti-Goat HRP-DAB Cell&Tissue Staining Kit (R&D system, Minneapolis, MN, USA) and counterstained with hematoxylin.

### 2.6. TUNEL Staining

Apoptotic tubular cells in renal I/R injury were detected using the terminal deoxynucleotidyl transferase-mediated deoxyuridine triphosphate (dUTP) nick-end labeling (TUNEL) method following the manufacturer's protocol (Roche Diagnostics, Mannheim, Germany). To calculate the apoptotic index (AI), the number of TUNEL-positive versus total cell nuclei was counted in the renal cortex and cortical-medullary junction in a blinded manner using 10 sequentially selected fields for each section containing at least 500 cells at 400x magnification. TUNEL-positive apoptotic cells exhibited brown nuclear staining. The AI was calculated by dividing the number of apoptotic cells by the total number of cells and multiplying by 100.

### 2.7. Electron Micrographs

The tissue samples (~1 mm thick) were fixed with 1 ml of 2.5% glutaraldehyde in phosphate buffer (pH 7.4) for 4 h and then were immersed in 1% osmium tetroxide for 2 h. The samples were washed in PBS three times for 10 min each, dehydrated in a graded series of ethanol, and imbibed in epoxy resin. Ultrathin sections (50–90 nm) were obtained using an ultramicrotome and were then poststained with uranyl acetate and lead citrate. The grids were examined using transmission electron microscopy (Tenai G2 Spirit; Hillsboro, OR, USA). For quantification, 10 fields of low magnification (×1000) were randomly obtained for each renal sample, and the total number of autophagic vacuoles was counted.

### 2.8. Renal Cell Culture and Treatment

**Figure 1:**
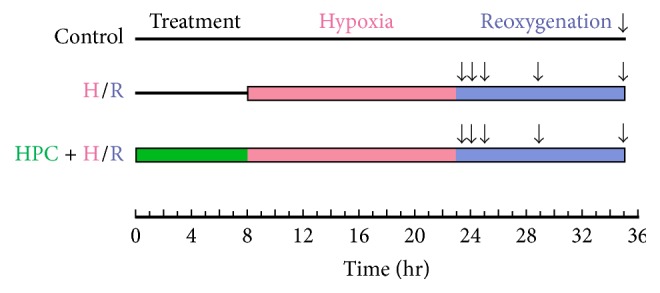
Hypoxia/reoxygenation (H/R) injury protocol. Control: cells were cultured constantly under normal conditions. H/R: cells received 15 h of oxygen and glucose deprivation (OGD) and 30 min, 1 h, 2 h, 6 h, or 12 h of reoxygenation in normal complete medium. Hypoxic preconditioning (HPC): transient OGD for 6 h and subsequent reoxygenation for 2 h in normal complete medium before prolonged H/R injury.

Human renal proximal tubular epithelial cells (HK-2; ATCC, Manassas, VA, USA) were isolated and cultured in Dulbecco's modified Eagle's growth medium (DMEM; ThermoFisher, Hudson, NH, USA) containing 10% fetal bovine serum under 5% CO_2_ and 95% atmospheric air at 37°C. To establish a hypoxia/reoxygenation (H/R) model* in vitro*, renal cells were subjected to oxygen and glucose deprivation (OGD) by changing the medium to serum/glucose-free DMEM and then were incubated in the Modular Incubator Chamber (MIC-101; Billups-Rothenberg, Del Mar, CA, USA) for 15 h in an atmosphere of 1% O_2_, 5% CO_2_, and 94% N_2_ at 37°C, followed by reoxygenation in normal complete medium and normoxic condition for 30 min, 1 h, 2 h, 6 h, or 12 h. Transient OGD for 6 h and subsequent reoxygenation for 2 h were implemented before prolonged H/R injury to achieve HPC. Control cells were cultured under normal condition. The experimental design is detailed in Figure 1.

### 2.9. Cell Viability Assay

Cell viability was detected with a Cell Counting Kit-8 (CCK-8, Dojindo, Kumamoto, Japan) according to the manufacturer's instructions. The absorbance was measured using a microplate reader at a wavelength of 450 nm. The percentage of living cells was calculated based on the ratio of absorbance of the experimental group to that of the normoxic group.

### 2.10. Annexin V-FITC/PI Staining

The cells were collected and incubated with 100 *μ*l of 1x binding buffer containing 5 *μ*l of Annexin V-FITC and 5 *μ*l of PI (ThermoFisher, Hudson, NH, USA). After a 30 min incubation in the dark at room temperature, the cells were analyzed by flow cytometry (BD FACSCalibur™, Becton Dickinson, NJ, USA). Annexin V+/PI− cells were considered early apoptotic cells.

### 2.11. Evaluation of Autophagy Flux

Primary HK-2 cells were infected with adenovirus encoding mRFP-GFP-LC3 (Hanbio, Shanghai, China) at a concentration of 20 multiplicity of infection. Twelve hours after adenovirus transduction, the cells were subjected to different interventions as described above. The cells were then visualized using a confocal microscope (Olympus, Tokyo, Japan). The processing and analysis of images were done using Metamorph (Molecular Devices) and the NIH ImageJ software. The number of GFP and mRFP dots was determined by manual counting of fluorescent puncta. At least 40 cells were scored in each group. The number of dots/cell was obtained by dividing the total number of dots by the number of nuclei in each microscopic field.

### 2.12. Statistical Analyses

The results are expressed as the mean ± standard deviation (SD). Two-tailed unpaired Student's *t*-tests were used to evaluate the data, and multiple comparisons among groups were performed using one-way analysis of variance (ANOVA) with post hoc Bonferroni test correction (SPSS, version 17.0). The level of statistical significance was *P* < 0.05.

## 3. Results

### 3.1. Ischemic Preconditioning Affects Autophagic Activity during Renal Ischemia/Reperfusion Injury

**Figure 2:**
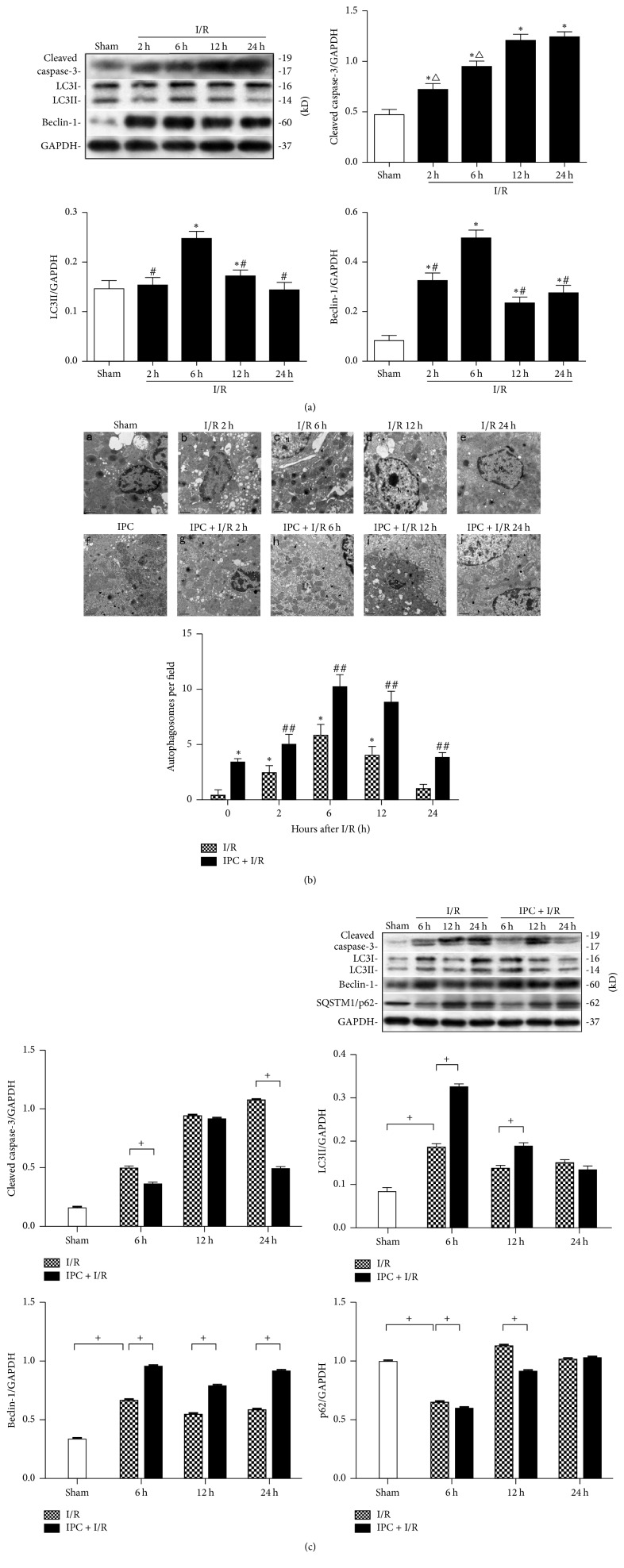
Autophagy induction during ischemia/reoxygenation (I/R) injury in rats with ischemic preconditioning (IPC). Male Sprague-Dawley rats were subjected to sham operation or 40 min of ischemia followed by 2 h, 6 h, 12 h, or 24 h of reperfusion with or without prior IPC, which consisted of four cycles of 8 min of clamping the left renal artery separated by 5 min of reperfusion. (a) Representative western blot gel documents and summarized data showing the levels of cleaved caspase-3, LC3II, and Beclin-1 protein expression. (b) Representative ultrastructure and semiquantification of autophagic vacuole formation in each group. N: nucleus; black arrowhead: autophagosome. Magnification ×20,000, scale bar = 1 *μ*m. (c) Representative western blot gel documents and summarized data showing the levels of cleaved caspase-3, LC3II, Beclin-1, and SQSTM/p62 protein expression. The data are expressed as the mean ± SD. *n* = 3 per group. ^*∗*^*P* < 0.05 versus the sham group. ^#^*P* < 0.05 versus the I/R 6 h group. ^△^*P* < 0.05 versus the I/R 24 h group. ^##^*P* < 0.05 versus the I/R group at the corresponding time point. ^+^*P* < 0.05.

Autophagy is a dynamic cellular catabolic process during renal I/R that is accompanied by apoptotic changes. Apoptosis during renal I/R was notably increased, as indicated by a gradual increase in cleaved caspase-3 (Figure 2(a)), which reached a maximum at 24 h after reperfusion. As indicated by the expression of LC3II and Beclin-1 in Figure 2(a), a basal level of autophagy was observed in the sham group, and it increased remarkably between 2 h and 6 h after reperfusion, peaked at 6 h, and then began to decline.

Autophagy is thought to be influenced by IPC in AKI. As shown in Figure 2(b), although autophagic vacuoles were rarely seen in the untreated sham kidneys (panel (A)), typical autophagosomes containing intracellular organelles (black arrowheads) were apparent after I/R and were increased compared to the sham kidneys at the different reperfusion time points, reaching a maximum at 6 h after reperfusion (panel (B)–(E)). However, the number of autophagosomes was further increased in the IPC-treated animals after I/R injury compared with that in the animals that underwent I/R injury alone at corresponding reperfusion time (*P* < 0.05, panel (F)–(J)). The quantitative analysis of the autophagic vacuoles confirmed the effects of IPC. In addition, the Atg immunoblots showed a dynamic change in the autophagosomes. The levels of LC3II and Beclin-1 were significantly increased in the IPC + I/R groups compared with those in the I/R groups after 6 h, 12 h, and 24 h of reperfusion (*P* < 0.05). Along with these changes, SQSTM1/p62 levels reached the nadir at I/R 6 h, which presented opposite change with the extension of reperfusion time. IPC pretreatment increased SQSTM1/p62 degradation at 6 h and 12 h after reperfusion (*P* < 0.05). But the expression of cleaved caspase-3 was higher at 6 h and 24 h after reperfusion in the animals that did not undergo IPC (*P* < 0.05, Figure 2(c)).

### 3.2. Ischemic Preconditioning Protects Renal Tissues from Ischemia/Reperfusion Injury

**Figure 3:**
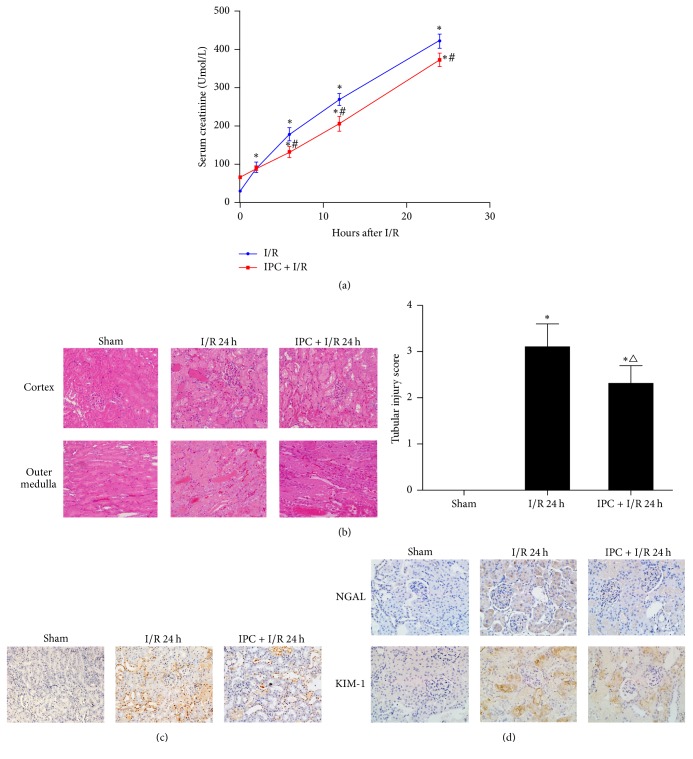
Ischemic preconditioning (IPC) alleviated renal ischemia/reoxygenation (I/R) injury. Male Sprague-Dawley rats were subjected to sham operation, IPC only, or 40 min of ischemia followed by 2 h, 6 h, 12 h, or 24 h of reperfusion with or without prior IPC. (a) Serum creatinine (Scr) concentrations. (b) Representative images of renal histology and quantitative analysis of tubular injury. Hematoxylin-eosin (H&E) staining. Representative slides of each group; ×200 magnification. (c) Representative images and quantitative data of terminal deoxynucleotidyl transferase-mediated nick-end labeling- (TUNEL-) positive nuclei in the kidney sections of each group. TUNEL staining; ×200 magnification. (d) Representative immunohistochemical images of tubular lesion in each group. ×400 magnification. KIM-1, kidney injury molecule-1; NGAL, neutrophil gelatinase-associated lipocalin. The data are expressed as the mean ± SD. *n* = 5 per group. ^*∗*^*P* < 0.05 versus the sham group. ^#^*P* < 0.05 versus the I/R group at the corresponding time point. ^△^*P* < 0.05 versus the I/R 24 h group.

To determine whether IPC protects renal tissues from I/R injury, we assessed the severity of renal damage using serum creatinine levels and renal pathology. Serum creatinine activity in the sham group remained at basal levels. IPC alone increased serum creatinine slightly; however, prior IPC ameliorated I/R injury as determined by the serum creatinine levels after 6 h, 12 h, and 24 h compared to the I/R only group (*P* < 0.05, Figure 3(a)). The results of H&E staining in the cortex and outer medulla showed extensive cell necrosis, loss of the brush border, cast formation, tubule dilatation expansion, and tubular cell degeneration after 40 min of ischemia and 24 h of reperfusion. Intervention with IPC significantly alleviated the observed pathological changes (*P* < 0.05, Figure 3(b)). TUNEL staining confirmed that there was no apparent apoptosis in the sham group, whereas the I/R and IPC + I/R groups showed increased apoptosis. However, the level of apoptosis was much lower in the IPC + I/R group than the I/R only group (*P* < 0.05, Figure 3(c)). Moreover, as the early biomarkers of AKI, the renal immunochemistry of NGAL and KIM-1 revealed that there were more intense KIM-1 and NGAL-positive areas in renal tubules of I/R group than IPC + I/R group (Figure 3(d)).

### 3.3. Hypoxia Preconditioning Affects Time-Dependent Autophagy and Apoptosis in Renal Hypoxia/Reoxygenation Injury

**Figure 4:**
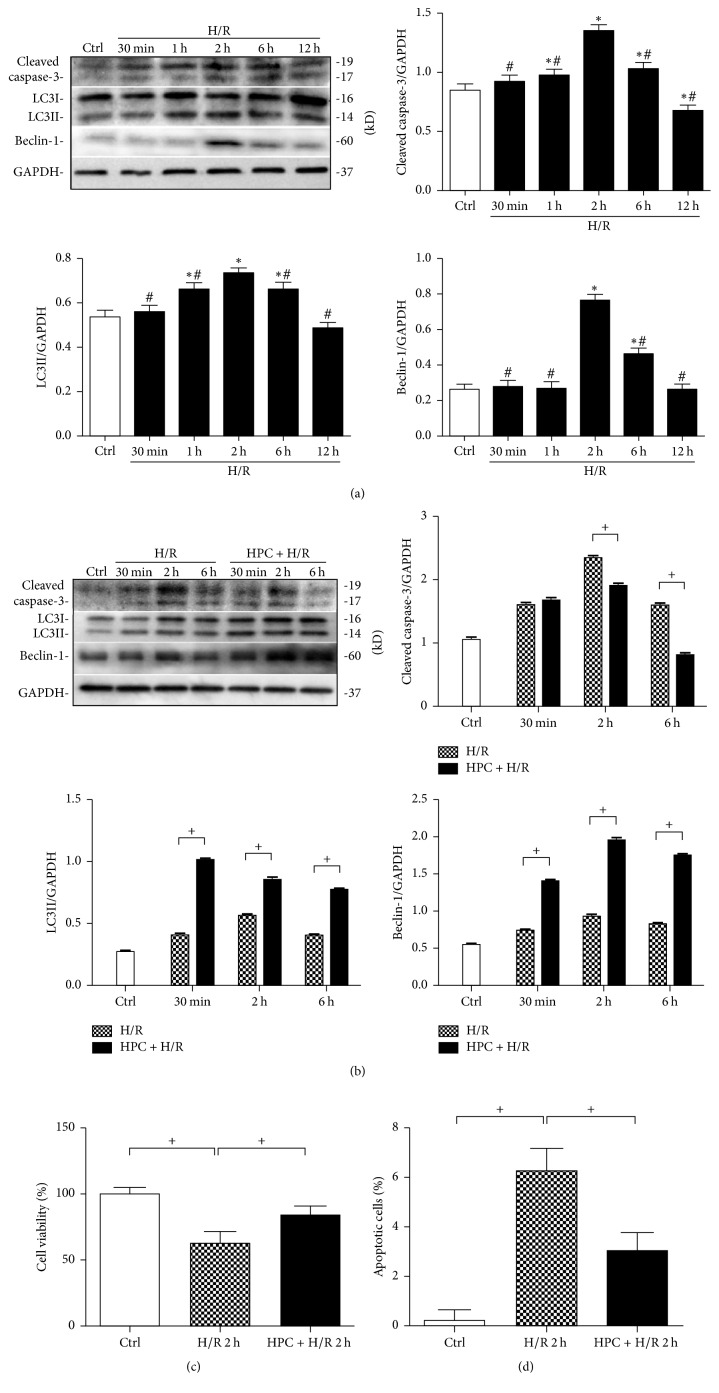
Hypoxic preconditioning (HPC) induced autophagy and reduced apoptosis in renal tubular cells. HK-2 cells were cultured under normal conditions or oxygen and glucose deprivation (OGD) for 15 h followed by reoxygenation in normal complete medium for 30 min, 1 h, 2 h, 6 h, and 12 h with or without prior HPC, which consisted of OGD for 6 h and subsequent reoxygenation for 2 h. ((a) and (b)) Representative western blot gel documents and summarized data showing the levels of cleaved caspase-3, LC3II, and Beclin-1 protein expression. (c) Determination and quantitative analysis of apoptotic cells by Annexin V-propidium iodide FACS analysis. (d) Renal tubular cell viability was evaluated by CCK-8 assay. The data are expressed as the mean ± SD. *n* = 3 per group. ^*∗*^*P* < 0.05 versus the control group. ^#^*P* < 0.05 versus the H/R 2 h group. ^+^*P* < 0.05.

Different durations of reoxygenation led to time-dependent changes in autophagy and apoptosis in the HK-2 cells exposed to H/R, which peaked at 2 h of reoxygenation, as indicated by the expression of LC3II, Beclin-1, and cleaved caspase-3 (*P* < 0.05, Figure 4(a)). HPC-tubular cells showed a significant time-dependent increase in the expression of LC3II and Beclin-1 and a decrease in the level of cleaved caspase-3 compared to the H/R group (*P* < 0.05, Figure 4(b)), suggesting that HPC promotes autophagic activity and inhibits apoptosis in renal cells exposed to H/R injury in a time-dependent manner. Apoptosis was then measured by Annexin V-fluorescein isothiocyanate (FITC)/propidium iodide (PI) staining and flow cytometric analysis. As shown in Figure 4(c), H/R resulted in an increased percentage of apoptotic cells (*P* < 0.05), which was suppressed by HPC pretreatment (*P* < 0.05). The CCK-8 assay showed that HK-2 cell viability was also diminished after H/R injury (*P* < 0.05, Figure 4(d)), whereas HPC increased cell viability at H/R 2 h (*P* < 0.05 versus H/R), indicating that hypoxia/reoxygenation (H/R) results in renal injury and HPC is involved in the protective effect against renal H/R injury and apoptosis.

**Figure 5:**
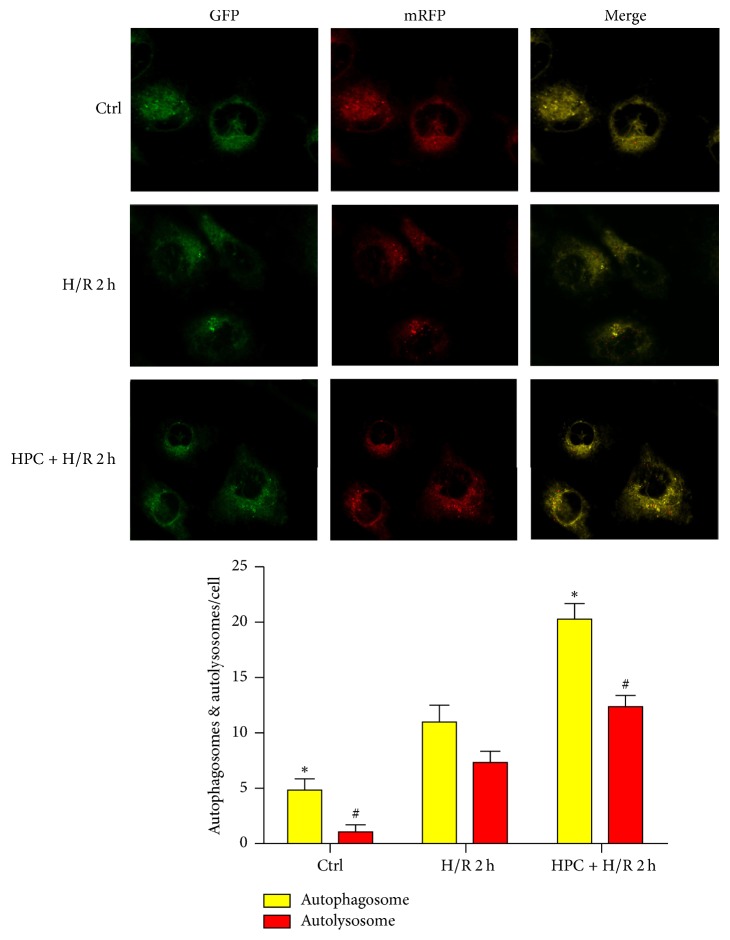
Hypoxic preconditioning (HPC) promoted autophagic flux in renal hypoxia/reoxygenation (H/R) injury. Representative images showing LC3 staining in renal tubular cells of different groups infected with mRFP-GFP-LC3 adenovirus for 12 h and then exposed to H/R injury with or without HPC prior to it. The autophagosomes (APs) were represented by yellow puncta and autolysosomes (ALs) were represented by red puncta in merged images. The results were obtained from three independent experiments with at least 100 cells analyzed. The data are expressed as the mean ± SD. ^*∗*^*P* < 0.05 versus the number of APs in the H/R 2 h group. ^#^*P* < 0.05 versus the number of ALs in the H/R 2 h group.

Given that autophagy flux is a dynamic process, autophagosome formation only could not fully reflect the extent of autophagic activity. We utilized the tandem RFP-GFP-LC3 adenovirus construct to monitor autophagic flux, according to previously described methods [11], because this technique is based on the pH difference between the acidic autolysosome and the neutral autophagosome. A green fluorescent protein (GFP) signal is quenched in the acidic and/or proteolytic conditions of the lysosome lumen. By contrast, a red fluorescent protein (RFP) is relatively stable. Therefore, colocalization of both GFP and RFP fluorescence implies a compartment that has not fused with a lysosome, such as a phagophore or autophagosome (green and red dots overlaid in merged images appear yellow), whereas autolysosomes are labeled red. Therefore, autophagy flux can be monitored by detecting the different fluorescence signals. Renal cells cultured in the normoxic environment displayed minimal autophagy flux. H/R increased the number of autophagic structures, showing an increase in both autophagosomes and autolysosomes (*P* < 0.05, Figure 5). Treatment with HPC increased the number of red and yellow puncta compared to the H/R group (*P* < 0.05), further confirming that HPC promoted the whole autophagy flux during renal H/R injury, rather than blocking lysosomal degradation.

## 4. Discussion

Although a large number of studies have confirmed autophagy induction during renal I/R, studies investigating the role of autophagy in AKI have reported both beneficial and detrimental effects [12].

It is generally recognized that autophagy could not only play a role in cell protection but also promote cell death. On the one hand, it is involved in degrading abnormal proteins, helping to prevent the accumulation of harmful substances and playing the role of cell scavenger. On the other hand, too high or too low levels of autophagy could damage organelles and turn them to autophagic cell death, which regulates cell survival or death through changing its threshold level [13, 14]. The regulatory mechanism of autophagy during AKI is still not clear, as it is reported that some signaling molecules such as Draper, JNK, and DAPK could direct autophagy to transform from promoting cell survival to cell death [15]. This may be related to the nonspecific destruction of macromolecular materials in cells or selective degradation of cytoprotective factors, leading to irreversible loss of cell viability [16]. Of note, Kimura and his colleagues demonstrated heightened renal I/R injury in proximal tubule-specific Atg5 knockout mice, providing the first* in vivo* genetic evidence for a renoprotective role in this AKI model [5].

While both autophagy and apoptosis can be induced in response to a common stimulus, autophagy contributes to cell survival by inhibiting necrosis or apoptosis in renal I/R. The activation of autophagy inhibits apoptosis by clearing misfolded/unfolded proteins and damaged organelles and mitochondria, inhibiting caspase-8 activation, as well as eliminating SQSTM1/p62 [17]. Caspases also play important roles in the regulation of autophagy that are separate from their roles in apoptosis. The activation of autophagy is an immediate response, whereas caspases are subsequently activated [18, 19]. Studies have demonstrated that autophagosomal membranes serve as platform for intracellular death-inducing signaling complex- (iDISC-) mediated caspase-8 activation and apoptosis [17]. Activated caspases cleared several key autophagy proteins including Beclin-1, Atg5, VPS34, ATG3, ATG4D, and Atg16 L, resulting in the suppression of autophagy [20]. It is known that autophagy is a double-edged sword in cell injury. On the one hand, it is involved in degradation of abnormal proteins to prevent the accumulation of harmful substances, which plays the role of cell scavenger. On the other hand, it acts as a switch which regulates life and death of cells through changes in the level of the threshold; it will damage organelles and transform cells to autophagic cell death (autophagic cell death, AuCD) if the level of autophagy is too high or too low, being the type II programming cell death [13]. As there are common signaling pathways and interplay regulatory mechanisms between autophagy and apoptosis, the duality of autophagy may be related to the regulation of apoptosis under stress condition. In our study, autophagy was observed in a dynamic manner both* in vivo* with renal I/R and* in vitro* with H/R. The expression of LC3II and Beclin-1 gradually increased before they were reduced as the duration of renal I/R increased, reaching a peak at 6 h after reperfusion. In addition, the expression of cleaved caspase-3 was low at the beginning of the reperfusion phase but increased as the reperfusion time extended. At the later reperfusion time, decreased autophagy might limit the ability of cells to remove damaged organelles and proteins, thus further aggravating the degree of apoptosis and slowing down recovery from I/R injury, although the expression of cleaved caspase-3 was notably decreased at 12 h after reoxygenation* in vitro*, which may be due to a lack of synthetic biological materials (Figure 4(a)). These results implied that early activation of autophagy inhibits the occurrence of apoptosis. As the apoptotic process improved, it inhibited autophagic activity in turn, suggesting that the autophagy and apoptotic cell death signaling pathways may interact with each other during AKI.

Autophagy is induced under stressful conditions to ensure cell survival by limiting necrosis or apoptosis. Similarly, decreased apoptosis has been reported as a biologically protective mechanism triggered by IPC [21, 22]. Recently, growing evidence has indicated that autophagy partly mediates IPC-induced organic protection by inhibiting apoptosis [22]. The cytoprotective role of IPC-induced autophagy has been reported in chemotherapy-treated livers, neuroblastoma cells, and human recipients of fatty liver grafts. Autophagy is also known to render cells resistant to apoptosis induced by topoisomerase II inhibitors [3, 23]. Zhang et al. [24] reported recently that HPC reduced myocardial cell injury by I/R through inducing FUNDC1-dependent mitophagy in Platelet. In this study, we clearly demonstrated that HPC induced autophagy flux and reduced apoptosis at different time points after renal H/R* in vitro*. In addition, we confirmed in the other studies that 3-MA inhibited IPC-induced protection against renal cell apoptosis and injury in renal I/R* in vitro *(data not shown), validating that IPC-mediated activation of autophagy is crucial in affording protection in renal I/R-induced AKI. Although the exact mechanism by which IPC-induced autophagy protects from oxidants, ATP depletion, or I/R injury is unclear, it is likely that autophagy activation meets necessary bioenergetic needs and eliminates protein aggregates and damaged organelles formed during the injury. Thus, a normal flux of autophagic activity is important for suppressing cell death [3]. Autophagic flux refers to complete autophagic activity with the degradation and removal of autophagic cargo. To monitor the various stages of autophagy during renal I/R, the tandem GFP-RFP-LC3 adenovirus construct was used in this study, confirming the effect of IPC on promoting the whole autophagy flux in renal I/R injury. Some situations including impairment in the autophagy process or overdigestion of cytoplasmic contents due to excessive autophagy may result in autophagic cell death [25]. IPC was reported to inhibit excessive autophagic cell death in a rat spinal cord I/R model [26]. Other studies have reported that autophagic cell death is motivated by prolonged IPC induced by cigarette smoke extract in human umbilical vein endothelial cells [27]. This discrepancy could result from differences in the methods and the degree of autophagy activation, the methods of implementing IPC, or test subjects.

In summary, we demonstrated that autophagy and apoptosis were altered in a time-dependent manner both in cultured RTECs and in kidney tissues. IPC could protect against I/R-induced renal cell apoptosis and injury by increasing autophagy. Further studies are needed to gain insights into the specific molecular mechanism whereby IPC mediates autophagy and protects cells from AKI.
